# Bacterial Community Structure after Long-term Organic and Inorganic Fertilization Reveals Important Associations between Soil Nutrients and Specific Taxa Involved in Nutrient Transformations

**DOI:** 10.3389/fmicb.2017.00187

**Published:** 2017-02-09

**Authors:** Fang Li, Lin Chen, Jiabao Zhang, Jun Yin, Shaomin Huang

**Affiliations:** ^1^Collaborative Innovation Center of Henan Grain Crops, Henan Agricultural UniversityZhengzhou, China; ^2^State Key Laboratory of Soil and Sustainable Agriculture, Institute of Soil Science, Chinese Academy of SciencesNanjing, China; ^3^Institute of Plant Nutrition and Environmental Resources Science, Henan Academy of Agricultural SciencesZhengzhou, China

**Keywords:** long-term fertilization, soil bacteria, specific taxa, co-occurrence, nutrient transformations

## Abstract

Fertilization has a large impact on the soil microbial communities, which play pivotal roles in soil biogeochemical cycling and ecological processes. While the effects of changes in nutrient availability due to fertilization on the soil microbial communities have received considerable attention, specific microbial taxa strongly influenced by long-term organic and inorganic fertilization, their potential effects and associations with soil nutrients remain unclear. Here, we use deep 16S amplicon sequencing to investigate bacterial community characteristics in a fluvo-aquic soil treated for 24 years with inorganic fertilizers and organics (manure and straw)-inorganic fertilizers, and uncover potential links between soil nutrient parameters and specific bacterial taxa. Our results showed that combined organic-inorganic fertilization increased soil organic carbon (SOC) and total nitrogen (TN) contents and altered bacterial community composition, while inorganic fertilization had little impact on soil nutrients and bacterial community composition. SOC and TN emerged as the major determinants of community composition. The abundances of specific taxa, especially *Arenimonas, Gemmatimonas*, and an unclassified member of *Xanthomonadaceae*, were substantially increased by organic-inorganic amendments rather than inorganic amendments only. A co-occurrence based network analysis demonstrated that SOC and TN had strong positive associations with some taxa (*Gemmatimonas* and the members of *Acidobacteria* subgroup 6, *Myxococcales, Betaproteobacteria*, and *Bacteroidetes*), and *Gemmatimonas, Flavobacterium*, and an unclassified member of *Verrucomicrobia* were identified as the keystone taxa. These specific taxa identified above are implicated in the decomposition of complex organic matters and soil carbon, nitrogen, and phosphorus transformations. The present work strengthens our current understanding of the soil microbial community structure and functions under long-term fertilization management and provides certain theoretical support for selection of rational fertilization strategies.

## Introduction

Fertilization is an essential agricultural practice used primarily to increase nutrient availability to crop plants, with concomitant changes in the soil properties, and microbial communities (Marschner et al., 2003). These changes can in turn influence plant growth and health by increasing soil nutrient turnover, plant disease suppression, or disease incidence, etc., Increasing the sustainability of cropping systems involves the reduced inputs of agrochemical fertilizers and combined organic amendments to facilitate biological interactions for the provision of plant nutrients (Lazcano et al., 2013). Of particular importance are soil microbial processes given their pivotal roles in the dynamics of soil carbon (C) and nitrogen (N) (Wardle et al., 1999).

Certain bacterial taxa at high taxonomic levels (e.g., phylum or class) can display properties of ecological coherence since they respond predictably to environmental variables (Philippot et al., 2010; Cederlund et al., 2014). Earlier, Fierer et al. (2007) proposed that certain bacterial phyla could be differentiated into the ecologically relevant copiotrophic (or r-selected) and oligotrophic (or K-selected) categories based on their substrate preferences and life strategies. As thus, long-term fertilization can directionally change the abundance of certain bacterial phyla. But we still have no sufficient understanding of soil bacterial taxa at low taxonomic levels (e.g., genus or species) in response to long-term fertilization. Long-term repeated addition of organic C seems to select for certain microbial taxa at low taxonomic levels that feed primarily on organic substrates and proliferate greatly, resulting in the changes in microbial community composition and soil nutrient status (Marschner et al., 2003; Zhong et al., 2010; Cederlund et al., 2014). As a consequence, specific microbial taxa of which the abundances are substantially increased by long-term fertilization should show some degree of connections with soil nutrients. Moreover, these taxa show potential beneficial or detrimental effects on crop productivity and even agroecosystem stability (Francioli et al., 2016). The complex associations occur between microbial taxa in the context of exogenous organics decomposition and soil nutrient transformations (Chen et al., 2015a; Banerjee et al., 2016). Network analysis of taxon co-occurrence, as measured by correlations between abundances of microbial taxa, can help decipher complex microbial association patterns and the ecological rules guiding community assembly (Barberán et al., 2012). Network analysis cannot only reveal inter-taxa associations in the shared niche spaces but also link microbial taxa to environmental parameters (Fuhrman, 2009; Barberán et al., 2012).

Recent studies have used high-throughput sequencing to provide new insights into the soil microbial diversity and community composition under long-term organic and inorganic fertilization (e.g., Lentendu et al., 2014; Calleja-Cervantes et al., 2015; Zhou et al., 2015; Chen C. et al., 2016; Ding et al., 2016; Francioli et al., 2016). However, less is known about which microbial taxa at low taxonomic levels are strongly influenced by long-term organic and inorganic fertilization and how these taxa are linked to soil nutrient parameters. To address these knowledge gaps, we selected a long-term field experiment receiving 24 years of various types of inorganic fertilizers and combined organics-fertilizers, measured the related parameters of soil nutrients, and analyzed the soil bacterial community characteristics using deep sequencing of the 16S rRNA gene amplicons. We used recently developed differential abundance analysis and network analysis of co-occurrence to unravel the potential effects of specific bacterial taxa and their associations with soil nutrients. Specifically, we examined: (i) whether combined organic-inorganic fertilization causes more pronounced shifts in the soil bacterial community composition than inorganic fertilization alone, (ii) which specific taxa are substantially stimulated by long-term fertilization, and (iii) which soil parameters are well linked to these taxa. We hypothesized that: since C and N are the most important resources for bacterial growth, soil C and N would show great associations with some specific taxa of which the abundances are substantially increased by long-term fertilization.

## Materials and methods

### Experimental description and sampling

A long-term fertilizer field experiment was established in 1990 at Zhengzhou (34°47′ N, 113°40′ E) of Henan Province, which is an important grain-producing area in China. The use of long-term field experiment has been approved by the legal entity “Henan Academy of Agricultural Sciences.” This region undergoes a temperate monsoon climate, with an average annual precipitation, and temperature of 641 mm and 14.4°C, respectively. The soil is a fluvo-aquic soil (clay 25%, sand 27%, an Inceptisol in the USDA soil taxonomy system) developing from alluvial sediments of the Yellow River (Chen L. et al., 2016). The experimental site included 33 plots (eleven treatments with three replicate plots of each, 10 × 4 m for each plot). All plots were randomly arranged and cement plates were inserted between plots. Except fertilization, all other management practices (e.g., irrigation, tillage, and pesticides) were the same for all plots. We selected seven treatments with application of various types of organics and fertilizers: MNPK (organic manure plus NPK fertilizers), SNPK (maize straw plus NPK fertilizers), HNPK (high rate of N fertilizer, regular PK fertilizers), LNPK (low rate of N fertilizer, regular PK fertilizers), NP (NP fertilizers), NK (NK fertilizers), and CK (unfertilized control). Urea (N 45%), superphosphate (P_2_O_5_ 12%), and potash (K_2_O 60%) were applied as NPK fertilizers. The cropping system was wheat (*Triticum aestivum* L.) and maize (*Zea mays* L.) rotation. 165.0 kg N ha^−1^ years^−1^, 82.5 kg P_2_O_5_ ha^−1^ years^−1^, and 82.5 kg K_2_O ha^−1^ years^−1^ were given at wheat season, and 187.5 kg N ha^−1^ years^−1^, 93.8 kg P_2_O_5_ ha^−1^ years^−1^, and 93.8 kg K_2_O ha^−1^ years^−1^ at maize season (except LNPK with 110.0 and 125.0 kg N ha^−1^ years^−1^ at wheat and maize seasons, respectively). Organic manure was cattle manure compost, on average, with N 12.7 g kg^−1^. Manure and straw were applied according to 7:3 of organic N:inorganic N ratio, i.e., 115.5 and 131.3 kg organic N ha^−1^ years^−1^ given at wheat and maize seasons, respectively, calculated from the N content of manure and straw.

The soils were sampled from the 0–20 cm plow layer in October 2014 after the harvest of maize. Six soil cores (5 cm diameter, 20 cm depth) were randomly collected from each replicate plot and pooled into one composite sample. After visible stones and plant residues were removed, soil was homogenized and passed through a 2 mm mesh. All samples were divided into three parts, one portion was air-dried to determine the general soil properties, one was stored at 4°C to measure the potential activities of C, N and P-acquiring enzymes, and one at −20°C for molecular analyses.

### Soil biochemical characterization

Soil pH was measured in a 1:2.5 soil solution (0.01 M CaCl_2_) with a Starter-2100 pH probe (Ohaus, Brooklyn, NY, USA). Soil organic C (SOC) and total N (TN) contents were determined by the K_2_Cr_2_O_7_ digestion and Kjeldahl determination methods, respectively. Available P (AP) content was determined by NaHCO_3_ extraction-colorimetry and available K (AK) content by CH_3_COONH_4_ extraction-flame photometry. Invertase activity (ITA) was analyzed using a 3,5-dinitrosalicylic acid method (Bandick and Dick, 1999). Urease activity (UEA) and alkine phosphatase activity (PTA) were quantified by measuring the breakdown rate of substrates urea and *p*-nitrophenyl-phosphate, respectively (Tabatabai, 1994).

### Preparation of amplicon library and sequencing

The total DNA was extracted from 0.50 g of fresh soils using the FastDNA Spin Kit for Soil (MP Biomedicals, Santa Ana, CA, USA), following the kit's directions. The isolated DNA was dissolved in 50 μl of TE buffer. DNA quality and concentrations were estimated based on spectrometry absorbance at wavelengths of 230, 260, and 280 nm detected by a NanoDrop ND-1000 spectrophotometer (NanoDrop Technologies, Wilmington, DE, USA). DNA was frozen at –80°C for downstream assays.

PCR amplification was carried out using primers F515 (5′-GTGCCAGCMGCCGCGGTAA-3′)/R806 (5′-GGACTACVSGGGTATCTAAT-3′) designed against the V4 region of the bacterial 16S rRNA gene (Caporaso et al., 2011). The reverse primer is barcoded with an 8-base sample-specific sequence to facilitate multiplexing of a sample set, and both primers contain sequencer adapter regions. The reaction mix was done in a volume of 50 μl consisting of 27 μl of ddH_2_O, 2 μl (5 μM) of each forward/reverse primer, 2.5 μl (10 ng) of template DNA, 5 μl (2.5 mM) of deoxynucleoside triphosphates, 10 μl of 5 × Fastpfu buffer, 0.5 μl of bovine serum albumin, and 1 μl of TransStart Fastpfu polymerase (TransGen, Beijing, China). Thirty thermal cycles (15 s at 94°C, 15 s at 55°C, and 30 s at 72°C) were conducted with a final extension at 72°C for 10 min. The quality of reaction products were verified on a 1% agarose gel.

PCR products were purified using a PCR Clean-up Purification Kit (MP Biomedicals), and quantified using a Qubit 2.0 fluorimeter (Invitrogen, Carlsbad, CA, USA). The purified amplicons were pooled in equimolar concentrations and loaded on a MiSeq Reagent Kit V2, and dual index sequencing of paired-end 250 bp was run on an Illumina MiSeq instrument (Illumina, San Diego, CA, USA). The sequence data were submitted to NCBI Sequence Read Archive (https://www.ncbi.nlm.nih.gov/sra/) with accession number SRP094809.

### Community bioinformatics and statistics

The clustering of operational taxonomic unit (OTU) was conducted using the UPARSE pipeline (Edgar, 2013), based on the following workflow: (i) quality filtering sequences using a “maxee” (i.e., maximum per sequence expected error frequency) value of 1 and trimmed to a consistent length; (ii) dereplicating identical sequences and removing singleton reads; (iii) building a *de novo* dataset of >97% similar sequence clusters and simultaneously removing chimera on this non-redundant dataset, using self-dataset and RDP gold sequence (Cole et al., 2014) as reference; (iv) generating OTU abundance table by mapping the total reads to representative sequence. Taxonomic annotation was assigned to each OTU representative sequence by UCLUST (Edgar, 2010) in QIIME v.1.9.0 (Caporaso et al., 2010) against the Greengenes 13_8 database. All sequences unassigned and assigned to archaea were removed.

The remaining sequences of all samples were rarefied to the same sequencing depth (25,223 sequences per sample). Principal coordinate analysis (PCoA) of the weighted and unweighted UniFrac (Lozupone and Knight, 2005) distances was calculated in the R package “ape.” Canonical analysis of principal coordinates (CAP) was performed in the R package “vegan.” When specifying CAP models, we constrained the analysis to edaphic factors while conditioning on all other factors. Effect significance of factors was calculated by running the vegan's permutest function over the CAP model using a maximum of 500 permutations. Mantel tests revealed the correlations between soil biochemical properties and bacterial community composition.

We used the R package “DESeq2” to calculate the OTUs differential abundance (i.e., log_2_-fold change in relative abundance of each OTU) for each fertilizer regime as compared to unfertilized control. Differential abundance analysis was conducted by fitting a generalized linear model with a negative binomial distribution to normalized value for each OTU and testing for differential abundance using a Wald test (Love et al., 2014). We adjusted *P*-values for multiple testing using the procedure of Benjamini and Hochberg (1995), and selected a false discovery rate (FDR) of 10% to denote statistical significance (Love et al., 2014; Whitman et al., 2016). Enriched and depleted OTUs were defined as OTUs with absolute differential abundance >1.0 and adjusted *P* < 0.1.

### Network analysis

Network analysis was conducted on bacterial OTUs and soil properties using the maximal information coefficient (MIC) in MINE software (Reshef et al., 2011). The MIC is a highly useful score that reveals the strength of linear and non-linear associations among variables (Reshef et al., 2011). To minimize pairwise comparisons and reduce network complexity, only OTUs with large differential abundance (adjusted *P* < 0.05) in at least one fertilizer regime were selected for network analysis. After the pairwise comparisons in MINE software, top 10,000 interactions were selected. The resulting 241 OTUs with strong positive (*r* > 0.8), strong negative (*r* < –0.8) and strong non-linear (MIC-ρ^2^ > 0.8) relationships were used for network construction in Cytoscape v.3.2.1 (Shannon et al., 2003). Network topological characteristics were calculated using NetworkAnalyzer tool in Cytoscape. Modular structure of highly interconnected nodes was analyzed using the MCODE application with default parameters. OTUs with maximum betweenness centrality scores were considered as keystone species (Vick-Majors et al., 2014; Banerjee et al., 2016).

## Results

### Soil biochemical properties

Soil pH and AP content showed no statistical differences between treatments. Inorganic fertilization (i.e., NK, NP, LNPK, and HNPK treatments) had little impact on SOC and TN contents. NPK fertilizers with combined application of manure (MNPK) and straw (SNPK) significantly increased SOC content by 52.3 and 47.1%, respectively, and significantly increased TN content by 36.4 and 49.1%, respectively. AK content was significantly enhanced by SNPK (56.6%), but little affected by other treatments (Table 1). Urease activity (UEA) was significantly improved by MNPK, but little affected by other treatments. Phosphatase activity (PTA) and invertase activity (ITA) showed 2.3 and 5.3-fold increases in SNPK, respectively, as compared to unfertilized control (Table 1).

**Table 1:** **Soil biochemical properties among different fertilization regimes**.

	**pH**	**SOC (mg g^−1^)**	**TN (mg g^−1^)**	**AP (μg g^−1^)**	**AK (μg g^−1^)**	**PTA (μg g^−1^ h^−1^)**	**UEA (μg g^−1^ h^−1^)**	**ITA (mg g^−1^ h^−1^)**
CK	8.11 ± 0.08a	6.16 ± 0.45b	0.55 ± 0.05c	8.27 ± 0.58a	96.70 ± 16.56bc	1.76 ± 0.28b	16.97 ± 3.05b	0.90 ± 0.31b
NK	8.27 ± 0.04a	5.99 ± 0.67b	0.61 ± 0.05bc	18.61 ± 19.33a	135.66 ± 7.24abc	2.02 ± 0.21b	20.03 ± 2.06ab	2.22 ± 1.18b
NP	8.12 ± 0.01a	7.59 ± 0.85ab	0.72 ± 0.03abc	11.54 ± 2.80a	92.83 ± 12.41c	1.43 ± 0.41b	20.25 ± 3.11ab	1.75 ± 0.90b
LNPK	8.18 ± 0.08a	7.88 ± 0.46ab	0.57 ± 0.04c	11.33 ± 2.57a	144.77 ± 21.95ab	1.68 ± 1.05b	17.01 ± 2.15b	2.26 ± 0.88b
HNPK	7.82 ± 0.34a	8.10 ± 0.77*ab*	0.64 ± 0.05abc	10.55 ± 5.38a	114.63 ± 2.70abc	2.02 ± 0.21b	22.08 ± 1.31ab	2.67 ± 0.58ab
MNPK	8.08 ± 0.00a	9.38 ± 1.03*a*	0.75 ± 0.05ab	12.29 ± 2.24*a*	123.42 ± 31.26*abc*	3.39 ± 0.99ab	23.94 ± 1.84a	2.53 ± 0.86b
SNPK	7.95 ± 0.27a	9.06 ± 1.69*a*	0.82 ± 0.14a	8.44 ± 0.82a	151.46 ± 14.17a	4.10 ± 1.20a	22.82 ± 0.40ab	4.81 ± 0.25a

*Means ± standard deviations (n = 3). Significant differences between means at α = 0.05 level detected by Tukey's HSD test are labeled with different letters. SOC, soil organic C; TN, total N; AP, available P; AK, available K; PTA, phosphatase activity; UEA, urease activity; ITA, invertase activity. CK, unfertilized control; LNPK, low N and regular PK fertilizers; HNPK, high N and regular PK fertilizers; MNPK, organic manure plus NPK fertilizers; SNPK, maize straw plus NPK fertilizers*.

### Relative abundance of major phyla and families

**Figure 1:**
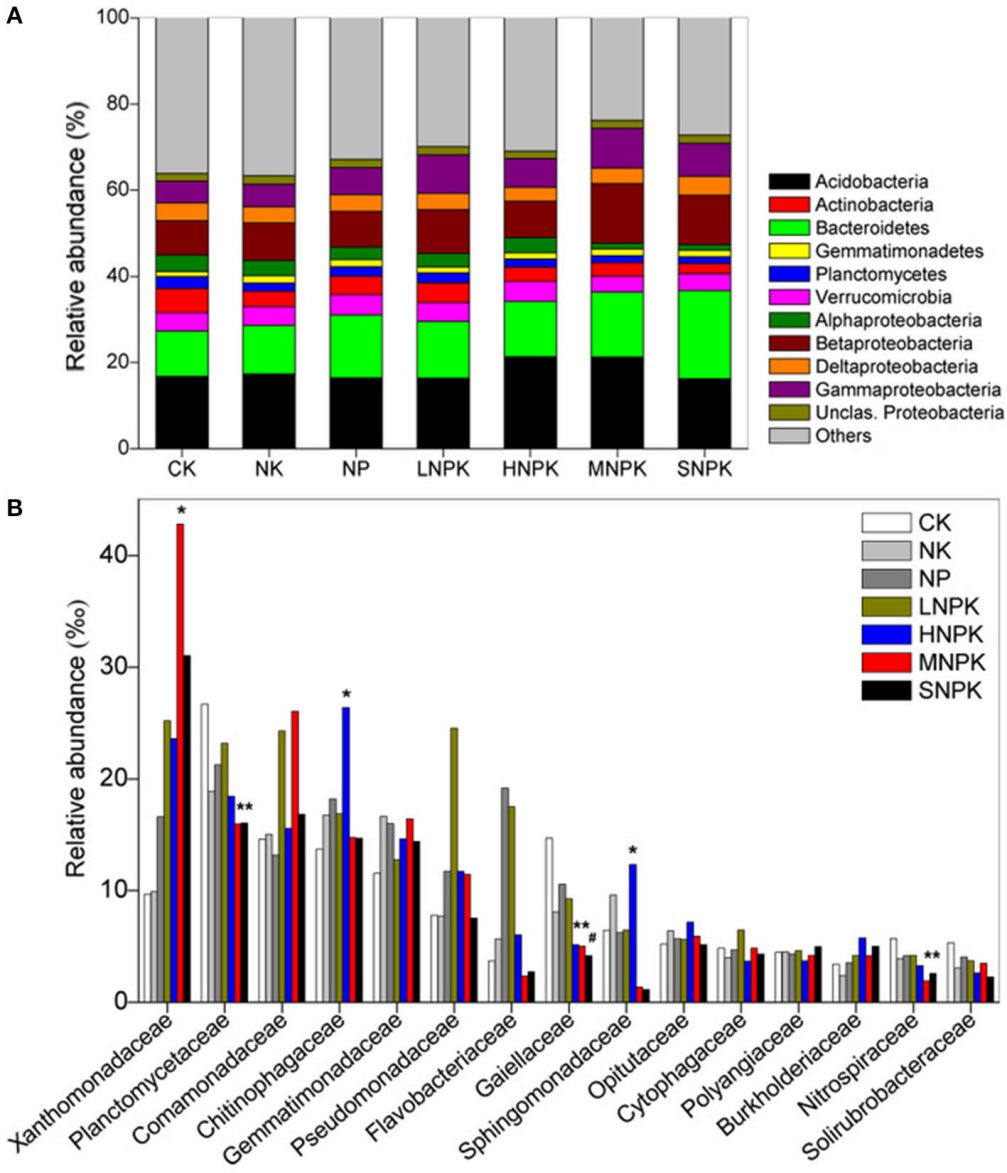
**Stacked and unstacked histograms showing the relative abundance of (A)** major bacterial phyla and dominant classes of *Proteobacteria* and **(B)** 15 most abundant bacterial families, respectively, in treatments CK (unfertilized control), NK, NP, LNPK (low rate of N, regular PK), HNPK (high rate of N, regular PK), MNPK (manure plus NPK) and SNPK (straw plus NPK). Each stripe represents the mean of three replicates. ^*^ and # mark significant differences at *P* < 0.05 and 0.01, respectively.

*Acidobacteria* (16–21%), *Bacteroidetes* (11–20%), and *Proteobacteria* (23–30%) were the dominant phyla across treatments. Among the classes of *Proteobacteria, Betaproteobacteria* (8–14%) had the highest relative abundance (Figure 1A). MNPK had higher relative abundance of *Betaproteobacteria*, and SNPK had higher *Bacteroidetes* than other treatments. *Acidobacteria* in HNPK and MNPK were more abundant (Figure 1A). Top 15 families with average relative abundance of >3.5% were analyzed (Figure 1B). MNPK led to a remarkable increase (4.4-fold increase) in the relative abundance of *Xanthomonadaceae*, but significant decrease in the relative abundances of *Planctomycetaceae, Gaiellaceae*, and *Nitrospiraceae*. Similarly, the relative abundances of *Planctomycetaceae, Gaiellaceae*, and *Nitrospiraceae* (especially *Gaiellaceae, P* < 0.01) were largely decreased by SNPK. The significantly increased abundances of *Chitinophagaceae* and *Sphingomonadaceae* occurred in HNPK (Figure 1B).

### Community structure, variation, and determinants

**Figure 2:**
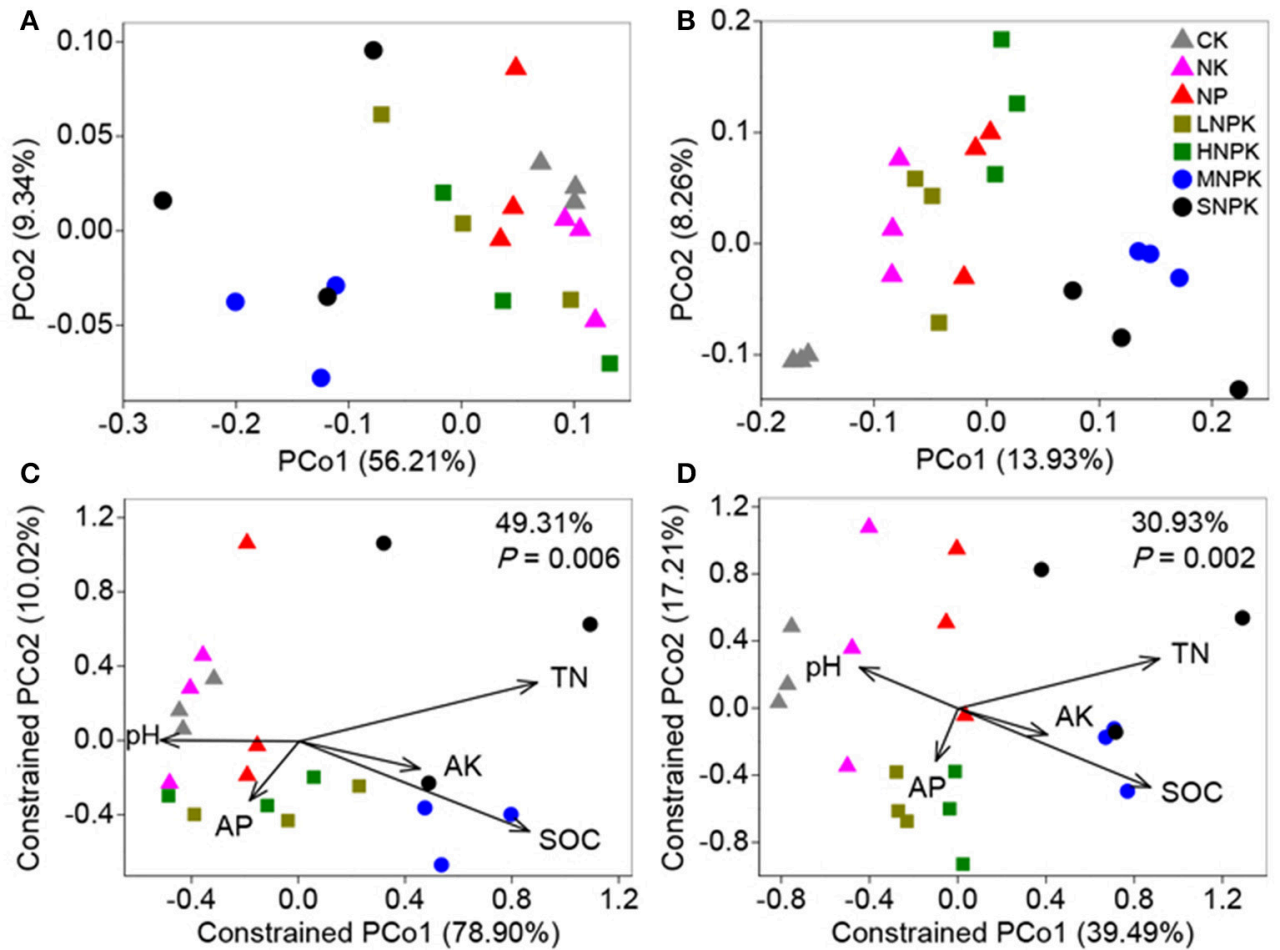
**Bacterial community variation between all samples from treatments CK (unfertilized control), NK, NP, LNPK (low rate of N, regular PK), HNPK (high rate of N, regular PK), MNPK (manure plus NPK) and SNPK (straw plus NPK). (A,B)** Principal coordinate analysis plots of OTU-based weighted **(A)** and unweighted **(B)** UniFrac distances between samples; **(C,D)** Canonical analysis of principal coordinates (CAP) of weighted **(C)** and unweighted **(D)** UniFrac distances quantifying the impacts of edaphic factors on bacterial community structure. CAP was constrained to the factors pH, SOC, TN, AP, and AK while conditioning on all other factors.

Principal coordinate analysis (PCoA) with weighted and unweighted UniFrac distance matrixes demonstrated the distinct community separation of MNPK and SNPK from other treatments, along the first principle coordinates (Figures 2A,B). The UniFrac distance is based on taxonomic relatedness, where weighted UniFrac takes abundance of taxa into consideration whereas unweighted UniFrac does not and is thus more sensitive to rare taxa. The moderate community separation between inorganic fertilization and non-fertilization (Figure 2B) indicates that the application of inorganic fertilizers has a certain influence on rare bacterial species. We used CAP to quantify the impacts of edaphic factors (i.e., pH, SOC, TN, AP, and AK) on bacterial community composition. The five constrained factors substantially contributed to bacterial community variation (49.31% of variation, *P* = 0.006, weighted UniFrac; 30.93% of variation, *P* = 0.002, unweighted UniFrac), and SOC and TN were the determinants among these factors (Figures 2C,D). Mantel test revealed great correlations of SOC (*P* ≤ 0.002) and TN (*P* = 0.001) with bacterial community composition (Table S1). SOC and TN also had significant correlations with the relative abundance of some major phyla and families, e.g., positive relationships with *Betaproteobacteria* and *Xanthomonadaceae*, and negative relationships with *Planctomycetes, Alphaproteobacteria*, and *Nitrospiraceae* (Table S2). These results suggest that the soil bacterial community composition under long-term fertilization was mainly driven by SOC and TN contents.

### Enriched and depleted OTUs by long-term fertilization

**Figure 3:**
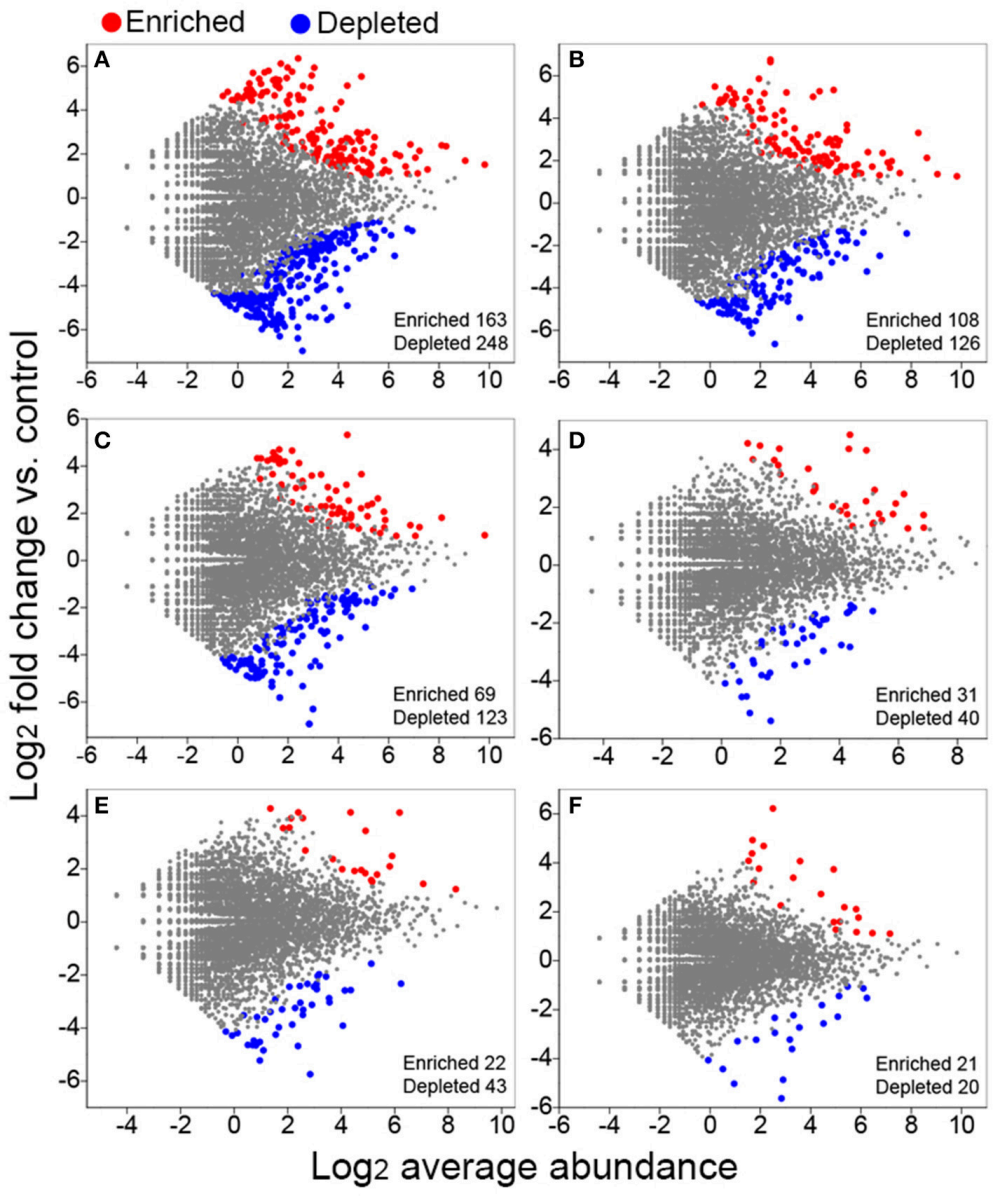
**Volcano plots illustrating OTUs significantly enriched (red) and depleted (blue) by long-term fertilization compared with unfertilized control as determined by differential abundance analysis**. Each point represents an individual OTU, and the Y axis indicates the abundance fold change vs. unfertilized control. **(A)** MNPK (manure plus NPK) vs. control; **(B)** SNPK (straw plus NPK) vs. control; **(C)** HNPK (high rate of N, regular PK) vs. control; **(D)** LNPK (low rate of N, regular PK) vs. control;**(E)** NP vs. control; **(F)** NK vs. control.

We conducted differential abundance analysis to identify OTUs that were strongly influenced by different fertilization regimes. Using OTU abundance from unfertilized soil as control and an adjusted *P*-value cutoff of 0.1, “enriched OTUs (eOTUs)” and “depleted OTUs (dOTUs)” specifically represent OTUs that increase and decrease significantly in relative abundance by more than doubling in response to long-term fertilization, respectively. There were 163 and 108 eOTUs (primarily the identifiable eOTUs from the phyla *Bacteroidetes, Betaproteobacteria, Gammaproteobacteria*, and *Acidobacteria*, Table 2), and 248 and 126 dOTUs (primarily phyla *Acidobacteria, Alphaproteobacteria, Actinobacteria*, and *Bacteroidetes*, Table 2) in MNPK and SNPK, respectively (Figures 3A,B; Dataset S1). Among top 10 most influential OTUs in MNPK and SNPK, eOTUs were mainly identified as *Arenimonas, Gemmatimonas*, and several unclassified members of *Xanthomonadaceae*, and dOTUs mainly as *Gaiella, Nitrospira, Sphingomonas*, and several unclassified members of *Sphingomonadaceae* (Table 2). There were much fewer OTUs enriched and depleted by inorganic fertilization compared to combined organic-inorganic fertilization, with the notable exception of HNPK in which 123 dOTUs (primarily phyla *Bacteroidetes, Actinobacteria*, and *Acidobacteria*, Table 2) were comparable to SNPK (Figures 3C–F; Dataset S1).

**Table 2:** **Differential abundance analysis exhibiting OTUs strongly influenced by MNPK (manure plus NPK), SNPK (straw plus NPK), and HNPK (high rate of N, regular PK) treatments, respectively**.

	**Enriched OTUs**					**Depleted OTUs**				
	**Phylum level**	**Count**	**Most influential OTUs**	**Log_2_ FC**	***P*****-adjusted**	**Phylum level**	**Count**	**Most influential OTUs**	**Log_2_ FC**	***P*** **adjusted**
MNPK vs. CK	Unclassified	46	*Arenimonas*	2.433	<0.001	Unclassified	91	*Gaiella*	−1.763	<0.001
	*Gammaproteobacteria*	28	*Arenimonas*	2.928	<0.001	*Acidobacteria*	34	*Gaiella*	−2.070	0.004
	*Bacteroidetes*	22	*Arenimonas*	2.755	<0.001	*Alphaproteobacteria*	21	*Nitrospira*	−3.079	0.002
	*Acidobacteria*	18	*Gemmatimonas*	2.971	<0.001	*Actinobacteria*	16	*Nitrospira*	−5.417	<0.001
	*Betaproteobacteria*	18	*Gemmatimonas*	5.020	<0.001	*Bacteroidetes*	16	Unclassified *Anaerolineaceae*	−4.012	0.007
	*Gemmatimonadetes*	11	*Lysobacter*	1.829	<0.001	*Deltaproteobacteria*	12	Unclassified *Chitinophagaceae*	−3.976	<0.001
	*Deltaproteobacteria*	6	Unclassified *Xanthomonadaceae*	4.051	<0.001	*Planctomycetes*	10	Unclassified *Comamonadaceae*	−1.573	0.011
	*Verrucomicrobia*	4	Unclassified *Xanthomonadaceae*	2.714	<0.001	*Verrucomicrobia*	10	Unclassified *Rhodospirillaceae*	−2.411	<0.001
	Others	10	Unclassified *Xanthomonadaceae*	2.545	<0.001	Others	38	Unclassified *Sphingomonadaceae*	−2.647	<0.001
	Total	163	Unclassified *Xanthomonadaceae*	5.114	<0.001	Total	248	Unclassified *Sphingomonadaceae*	−2.355	0.002
SNPK vs. CK	Unclassified	42	*Arenimonas*	2.352	<0.001	Unclassified	46	*Gaiella*	−1.754	0.009
	*Bacteroidetes*	20	*Arenimonas*	2.720	0.001	*Acidobacteria*	19	*Gaiella*	−3.369	<0.001
	*Gammaproteobacteria*	12	*Arenimonas*	3.427	<0.001	*Alphaproteobacteria*	14	*Rhodospirillales mumbers*	−2.797	<0.001
	*Betaproteobacteria*	10	*Aureispira*	3.111	0.004	*Actinobacteria*	9	*Nitrospira*	−4.468	<0.001
	*Acidobacteria*	9	*Gemmatimonas*	3.011	<0.001	*Bacteroidetes*	8	*Sphingomonas*	−1.886	0.032
	*Deltaproteobacteria*	6	*Gemmatimonas*	2.447	0.030	*Gammaproteobacteria*	5	Unclassified *Chitinophagaceae*	−6.649	0.002
	*Gemmatimonadetes*	5	*Gemmatimonas*	4.348	0.006	*Verrucomicrobia*	5	Unclassified *Rhodospirillaceae*	−2.024	0.027
	*Verrucomicrobia*	2	Unclassified *Cryomorphaceae*	3.691	0.022	*Deltaproteobacteria*	4	Unclassified *Sphingomonadaceae*	−2.410	0.002
	Others	2	Unclassified *Xanthomonadaceae*	2.170	0.022	Others	16	Unclassified *Sphingomonadaceae*	−2.588	0.002
	Total	108	Unclassified *Xanthomonadaceae*	5.266	<0.001	Total	126	Unclassified *Sphingomonadaceae*	−3.082	0.047
HNPK vs. CK	Unclassified	17	*Arenimonas*	2.200	0.001	Unclassified	55	*Gaiella*	−1.833	0.001
	*Bacteroidetes*	14	*Cellvibrio*	2.618	0.001	*Bacteroidetes*	11	*Gaiella*	−1.628	0.002
	*Gammaproteobacteria*	10	*Gemmatimonas*	2.059	<0.001	*Actinobacteria*	10	*Gaiella*	−2.154	0.007
	*Acidobacteria*	9	*Lacibacter*	2.590	0.009	*Acidobacteria*	9	*Gaiella*	−1.782	0.012
	*Gemmatimonadetes*	4	*Luteolibacter*	1.842	0.014	*Gammaproteobacteria*	6	*Nitrospira*	−6.931	<0.001
	*Verrucomicrobia*	4	*Sphingomonas*	1.389	<0.001	*Verrucomicrobia*	5	Unclassified *Chitinophagaceae*	−5.330	<0.001
	*Alphaproteobacteria*	3	Unclassified *Chitinophagaceae*	2.327	<0.001	*Betaproteobacteria*	4	Unclassified *Comamonadaceae*	−1.624	0.007
	*Betaproteobacteria*	3	Unclassified *Chitinophagaceae*	1.969	0.012	*Deltaproteobacteria*	4	Unclassified *Planctomycetaceae*	−4.833	0.028
	Others	5	Unclassified *Xanthomonadaceae*	2.953	0.006	Others	19	Unclassified *Rhodocyclaceae*	−4.743	0.036
	Total	69	Unclassified *Xanthomonadaceae*	5.321	<0.001	Total	123	Unclassified *Rhodospirillaceae*	−1.489	0.029

### Network associations among OTUs and soil properties

**Figure 4:**
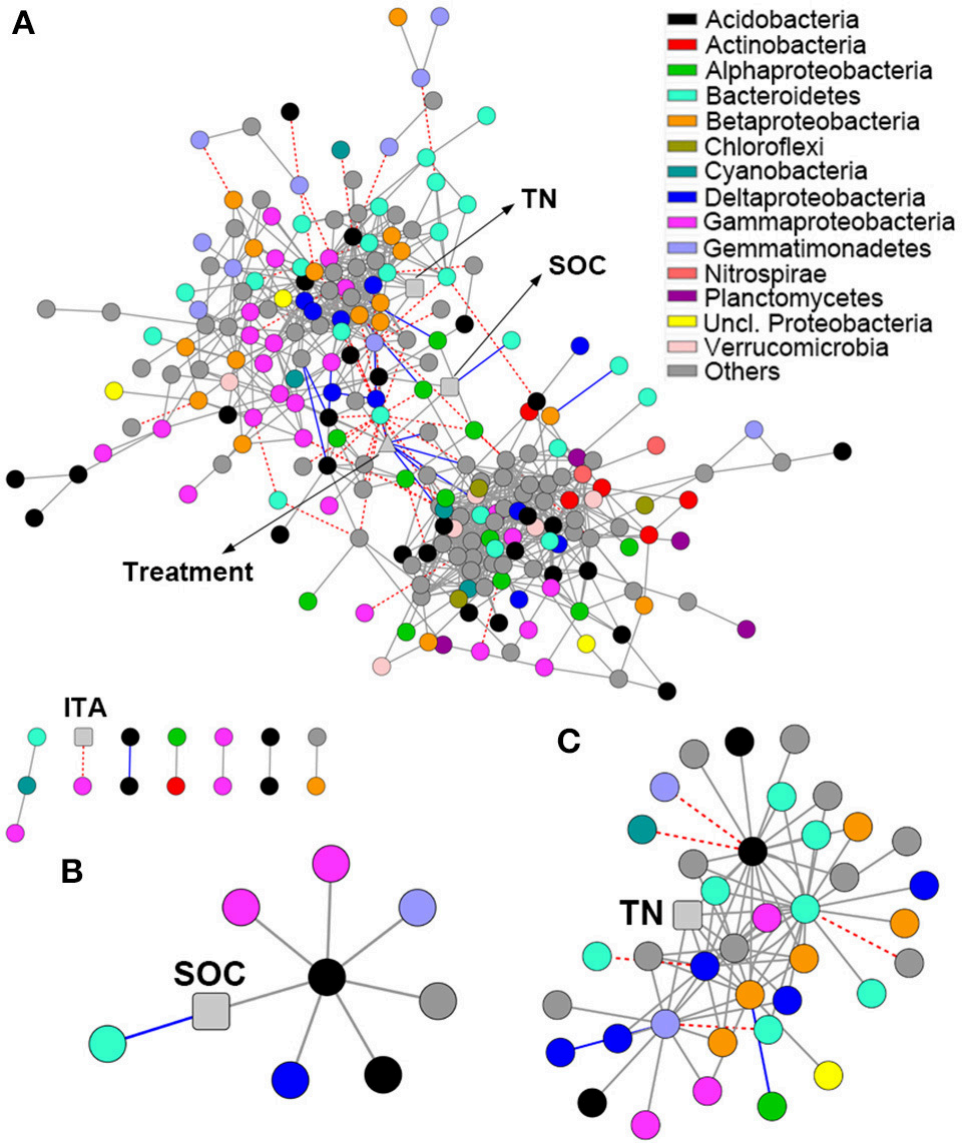
**Network analysis revealing the associations among bacterial OTUs and soil properties**. Gray solid line, blue solid line and red dash line represent strong positive linear (*r* > 0.8), strong negative linear (*r* < −0.8) and strong nonlinear (MIC-ρ^2^ > 0.8) relationships, respectively. Colored nodes signify corresponding OTUs assigned to major phyla and classes. Soil properties are indicated with round rectangle, and treatment with triangle. SOC, soil organic C; TN, total N; ITA, invertase activity. **(A)** Network co-occurrences of OTUs substantially enriched by long-term fertilization; **(B,C)**, Subnetworks for the associations of SOC **(B)** and TN **(C)**.

The network comprised 874 significant associations (edges) of 245 nodes, with an average clustering coefficient of 0.32 and overall diameter of 11 edges (Table S3). The network exhibited an average number of neighbors of 7.14 and characteristic path length of 3.99 (Table S3). Network edges were predominantly composed of strong positive associations, and the dominant identifiable OTUs belonged to *Acidobacteria, Bacteroidetes*, and *Gammaproteobacteria* (Figure 4A). SOC showed a strong positive association with one *Acidobacteria* subgroup 6 (Gp6) member (Figure 4B; Dataset S2). TN showed strong positive associations with *Gemmatimonas*, one *Acidobacteria* Gp6 member, one *Myxococcales* member and two members within *Betaproteobacteria* and *Bacteroidetes* (Figure 4C; Dataset S2). Based on betweenness centrality scores, the OTUs identified as keystone taxa were *Gemmatimonas, Flavobacterium* and one Subdivision3 member within *Verrucomicrobia* (Dataset S2).

## Discussion

We used deep 16S amplicon sequencing to investigate the bacterial community characteristics in the fluvo-aquic soil treated for 24 years with various types of inorganic fertilizers and combined organic amendments and inorganic fertilizers. Bacterial communities across all treatments were dominated by the phyla *Acidobacteria, Bacteroidetes*, and *Proteobacteria* (Figure 1), which roughly correspond to previous studies in agricultural soils (Zhong et al., 2015; Zhou et al., 2015; Ding et al., 2016). As anticipated, combined organic-inorganic fertilization dramatically changed the soil bacterial community composition, but inorganic fertilization alone had little impact on bacterial community composition (Figure 2). Similar results were reported previously, based on the phospholipid fatty acid analysis (Lazcano et al., 2013; Williams et al., 2013). Our study supports the principle that the bacterial community in cropland soils is primarily influenced by organic component of agricultural fertilization. Bacterial growth is often limited by C availability, even in soils with high C:N ratio (Demoling et al., 2007). Fast-growing copiotrophic bacteria proliferate soon after the supply of readily available C substrates to the soil and decrease later, and the growth of slow-growing oligotrophic bacteria recovers as substrate C availability declines over time. Thus, the succession of bacterial community occurs during repeated addition of organic matters. It was observed even that bacterial growth and community structure were changed in a short time period by a small fraction of organic component in the total amount of fertilizers applied (Lazcano et al., 2013).

There is the possibility that allochthonous inputs of bacterial taxa from organic amendments contribute to the alteration in the soil bacterial community composition. However, some studies have revealed the negligible effects of introduced bacteria from manure amendment on the soil bacterial community (Chu et al., 2007; Sun et al., 2015). The microbes in manure which are well adapted to the gut environments are less competitive than indigenous microbes in soils (Sun et al., 2015). *Bacteroidetes* is one of the most abundant bacterial phyla in cattle manure (Shanks et al., 2011), but we did not find large changes in the relative abundance of *Bacteroidetes* between treatments with and without cattle manure amendment (Figure 1). In addition, bacterial responses to manure amendment differ somewhat from straw amendment. We observed increased abundance of *Acidobacteria* but decreased abundance of *Bacteroidetes* in manure plus NPK treatment (MNPK) compared to straw plus NPK treatment (SNPK), and bacterial phylotypes were more enriched and depleted by MNPK compared to SNPK (Figures 1, 3). The possible explanation is that organic manure contains more labile organic C and lower C:N ratio than crop straw. The type of C input has been found to be a main factor determining the shifts in the soil bacterial community structure (Eilers et al., 2010; Shi et al., 2011; Pascault et al., 2013).

The results of ordination and correlation analyses between bacterial community characteristics and soil properties reveal that soil C and N contents are the main drivers for bacterial community composition under long-term fertilization (Figure 2; Tables S1, S2). When soil was amended with exogenous organics and fertilizers, certain microbial taxa are able to decompose organics and simultaneously acquire N from fertilizers to grow and reproduce rapidly under appropriate C:N stoichiometric ratios. In this situation, exogenous organics, and microbial metabolites are continuously decomposed and transformed, resulting in the changes in soil C and N contents over a long period of time. On the other hand, manure and straw amendments can stimulate the activity of some oligotrophs to mineralize recalcitrant soil organic matter (SOM) by using fresh organic matter as energy source, and cause a short-term change in SOM turnover, aka priming effect (Blagodatskaya and Kuzyakov, 2008). Therefore, soil C and N contents have necessary links with bacterial community composition under long-term fertilization. The importance of soil C and N contents in shaping bacterial community composition was also reported previously (Helgason et al., 2010; Shen et al., 2010; Sul et al., 2013; Liu et al., 2014; Chen C. et al., 2016).

We conducted differential abundance analysis to pick out OTUs that were responsible for the observed community differences between the fertilized and unfertilized soils. The OTUs primarily from *Bacteroidetes, Betaproteobacteria, Gammaproteobacteria*, and *Acidobacteria* were significantly enriched by long-term fertilization, especially combined organic-inorganic fertilization (Table 2). *Bacteroidetes, Betaproteobacteria*, and *Gammaproteobacteria* as copiotrophs thrive under conditions where substrate availability is high (Fierer et al., 2007; Eilers et al., 2010; Nemergut et al., 2010; Chen et al., 2015b). Despite there are many oligotrophic members within the *Acidobacteria* phylum (Nemergut et al., 2010; Pascault et al., 2013), some *Acidobacteria* members were depleted but some were enriched by combined organic-inorganic fertilization (Table 2). Our results are in agreement with previous findings that some *Acidobacteria* members (e.g., subgroups 1 and 7) were very few but some (e.g., subgroups 4 and 6) were abundant in soils with high content of organic C (Liu et al., 2014). We analyzed top 10 most influential OTUs at the genus level, and found that most enriched OTUs by manure and straw amendments were *Arenimonas, Gemmatimonas*, and several unclassified members of the *Xanthomonadaceae* family (Table 2). The *Arenimonas* species have catalytic activities of acid and alkaline phosphatase, esterase, esterase lipase, lipase, arylamidase, etc., (Jin et al., 2012; Huy et al., 2013; Makk et al., 2015). According to genome sequencing information, *Arenimonas* is capable of metabolizing casein, gelatin, β-hydroxybutyric acid, tyrosine, L-alaninamide, L-glutamic acid, and glycyl-L-glutamic acid (Chen et al., 2015c). *Gemmatimonas* is able to modulate C and N intakes according to their metabolic needs under various conditions (Carbonetto et al., 2014). *Gemmatimonas* shows high abundance in soils added with pyrogenic organic matters (Xu et al., 2014; Whitman et al., 2016), indicating that *Gemmatimonas* is likely to decompose polyaromatic C. *Gemmatimonas* was reported as a polyphosphate-accumulating bacterium (Zhang et al., 2003), and could be stimulated by increased input of P fertilizer in agricultural management (Su et al., 2015). These findings are supported by our results that some *Gemmatimonas* phylotypes (e.g., OTU_289 and OTU_78; dataset S1) were enriched by NP treatment rather than NK treatment. Moreover, *Gemmatimonas* was found at a high abundance in the rhizosphere of healthy wheat plants (Yin et al., 2013), indicating that *Gemmatimonas* may help suppress diseases and promote plant growth. The *Xanthomonadaceae* members within *Gammaproteobacteria* are known hydrocarbon decomposers, and they have also been shown to obtain C from co-occurring microorganisms (Lueders et al., 2006). Moreover, the *Xanthomonadaceae* family has been previously described as being dominant in the decomposing process of wood materials (Folman et al., 2008; Hervé et al., 2014). In summary, specific bacterial taxa substantially enriched by combined organic-inorganic fertilization play important roles in organics decomposition and soil C, N, and P transformations.

Since C and N are the most important resources for bacterial growth, soil C, and N would show great associations with some specific taxa significantly enriched by long-term fertilization. Our hypothesis is confirmed by a co-occurrence based network analysis that revealed strong positive associations of SOC and TN with some taxa (e.g., *Gemmatimonas* and the members of *Acidobacteria* subgroup 6 and *Myxococcales*) (Figure 4; Dataset S2). The roles of *Gemmatimonas* involved in soil nutrient transformations are discussed above. Some subgroups of *Acidobacteria* are abundant in soils with high SOC level (Liu et al., 2014), and their ability to decompose organic matters has been reported previously (Rawat et al., 2012; Tveit et al., 2014). *Myxococcales* members act as the active micropredators in the soil microbial food web and play important roles in soil C sequestration (Lueders et al., 2006; Zhou et al., 2014). Betweenness centrality score discerns the modules that are most important in maintaining connectivity in an ecological network, and thus can be used for identification of keystone species (Vick-Majors et al., 2014). Based on betweenness centrality score, *Gemmatimonas, Flavobacterium*, and an unclassified Subdivision3 member of *Verrucomicrobia* were identified as the keystone taxa. *Flavobacterium* is responsible for heterotrophic denitrification (Wang et al., 2016). *Verrucomicrobia* members have been previously reported as degraders of recalcitrant organic matters (Fierer et al., 2013).

In terms of organic and inorganic fertilization alone, the former usually produces lower crop yield (Seufert et al., 2012), but the latter causes more environmental problems (Davidson, 2009). The integrated strategies of organic amendments and inorganic fertilizers are evaluated as a most effective way to enhance crop productivity and increase SOM level in China (Gong et al., 2009; Liu et al., 2010; Wei et al., 2016). Our long-term observation data also shows comparable and even higher yields of maize and wheat under combined organic-inorganic fertilization compared to inorganic fertilization (Figure S1). Meanwhile, combined organic-inorganic fertilization increased the potential activities of soil invertase, urease, and alkaline phosphatase, which are three typical microbial exoenzymes involved in C, N, and P mineralization. More importantly, compared to inorganic fertilization, combined organic-inorganic fertilization enriched more amounts of specific bacterial taxa. These taxa are implicated in the decomposition of complex organic matters and soil nutrient transformations, and are thus beneficial for plant growth by improving nutrient availability.

## Author contributions

JZ, FL, and LC designed the study. LC analyzed the data and wrote the manuscript. FL collected and analyzed soil samples. JY and SH contributed to the management and maintenance of long-term field experiment. All authors reviewed the manuscript.

### Conflict of interest statement

The authors declare that the research was conducted in the absence of any commercial or financial relationships that could be construed as a potential conflict of interest.
