# Supplementary material for: Bacterial Community Structure after Long-term Organic and Inorganic Fertilization Reveals Important Associations between Soil Nutrients and Specific Taxa Involved in Nutrient Transformations
Source: Front Microbiol. 2017 Feb 9;8:187. doi: 10.3389/fmicb.2017.00187 (PMC5298992; doi:10.3389/fmicb.2017.00187)
Supplement: Supplementary file 2 [file DataSheet2.docx]

**Table S1** Mantel test revealing the correlations between soil biochemical properties and bacterial community composition (Bray-Curtis distance).

|  | Pearson’s correlation | |  | Spearman’s rank correlation | |  | Kendall’s rank correlation | |
| --- | --- | --- | --- | --- | --- | --- | --- | --- |
|  | r | *P* |  | r | *P* |  | r | *P* |
| pH | 0.131 | 0.154 |  | 0.069 | 0.266 |  | 0.052 | 0.218 |
| SOC | **0.389** | **0.002** |  | **0.346** | **0.002** |  | **0.238** | **0.001** |
| TN | **0.621** | **0.001** |  | **0.494** | **0.001** |  | **0.342** | **0.001** |
| AP | –0.092 | 0.699 |  | –0.168 | 0.958 |  | –0.115 | 0.950 |
| AK | 0.132 | 0.114 |  | 0.057 | 0.239 |  | 0.035 | 0.270 |
| **PTA** | **0.284** | **0.044** |  | **0.377** | **0.005** |  | **0.247** | **0.007** |
| **UEA** | **0.262** | **0.023** |  | **0.258** | **0.010** |  | **0.174** | **0.013** |
| **ITA** | **0.429** | **0.003** |  | **0.352** | **0.005** |  | **0.237** | **0.002** |

Bolds indicate significant correlations (*P* < 0.05).

SOC, soil organic C; TN, total N; AP, available P; AK, available K; PTA, phosphatase activity; UEA, urease activity; ITA, invertase activity.

**Table S2** The correlations of soil biochemical properties with the relative abundance of major phyla and families.

|  | pH | SOC | TN | AP | AK | PTA | UEA | ITA |
| --- | --- | --- | --- | --- | --- | --- | --- | --- |
| *Acidobacteria* |  |  |  |  |  |  |  |  |
| *Bacteroidetes* | –0.519^*^ |  | 0.731^***^ |  |  | 0.490^*^ |  | 0.595^**^ |
| *Verrucomicrobia* |  |  |  |  |  |  |  |  |
| *Actinobacteria* |  | –0.454^*^ | –0.596^**^ |  |  | –0.491^*^ | –0.598^**^ | –0.623^**^ |
| *Planctomycetes* |  | –0.524^*^ | –0.695^***^ |  |  | –0.490^*^ | –0.682^***^ | –0.568^**^ |
| *Gemmatimonadetes* |  |  |  | 0.484^*^ |  |  |  |  |
| *Alphaproteobacteria* |  | –0.665^***^ | –0.773^***^ |  |  | –0.642^**^ | –0.580^**^ | –0.572^**^ |
| *Betaproteobacteria* |  | 0.548^*^ | 0.456^*^ |  |  |  |  |  |
| *Deltaproteobacteria* |  |  |  |  |  |  |  |  |
| *Gammaproteobacteria* |  | 0.500^*^ |  |  |  |  |  |  |
| Unclas. *Proteobacteria* |  |  |  |  |  |  |  |  |
|  |  |  |  |  |  |  |  |  |
| *Xanthomonadaceae* | –0.464^*^ | 0.617^**^ | 0.543^*^ |  |  | 0.453^*^ | 0.466^*^ |  |
| *Planctomycetaceae* |  | –0.527^*^ | –0.705^***^ |  |  | –0.487^*^ | –0.683^***^ | –0.572^**^ |
| *Comamonadaceae* |  |  |  |  |  |  |  |  |
| *Chitinophagaceae* |  |  |  |  |  |  |  |  |
| *Gemmatimonadaceae* |  |  |  | 0.484^*^ |  |  |  |  |
| *Pseudomonadaceae* |  |  |  |  |  |  |  |  |
| *Flavobacteriaceae* |  |  |  |  |  |  |  |  |
| *Gaiellaceae* |  | –0.539^*^ | –0.545^*^ |  |  | –0.517^*^ | –0.671^***^ | –0.648^***^ |
| *Sphingomonadaceae* |  | –0.436^*^ | –0.573^**^ |  |  | –0.514^*^ |  |  |
| *Opitutaceae* |  |  |  |  |  |  |  |  |
| *Cytophagaceae* |  |  |  |  |  |  |  |  |
| *Polyangiaceae* |  |  |  |  |  |  |  |  |
| *Burkholderiaceae* | –0.508^*^ | 0.535^*^ |  |  |  |  |  |  |
| *Nitrospiraceae* |  | –0.692^***^ | –0.62^**^ |  |  | –0.482^*^ | –0.662^***^ | –0.528^*^ |
| *Solirubrobacteraceae* |  |  |  |  |  |  |  | –0.525^*^ |

*, ** and *** denote significant correlations at the levels of 0.05, 0.01 and 0.001, respectively.

SOC, soil organic C; TN, total N; AP, available P; AK, available K; PTA, phosphatase activity; UEA, urease activity; ITA, invertase activity.

**Table S****3** Network topological characteristics calculated by NetworkAnalyzer tool in Cytoscape v.3.2.1.

| Network topological characteristics | |
| --- | --- |
| Number of nodes | 245 |
| Number of edges | 874 |
| Network density | 0.03 |
| Network heterogeneity | 0.93 |
| Clustering coefficient | 0.32 |
| Connected components | 8 |
| Network diameter | 11 |
| Network radius | 1 |
| Network centralization | 0.09 |
| Shortest paths | 52688 |
| Characteristic path length | 3.99 |
| Avg. number of neighbors | 7.14 |


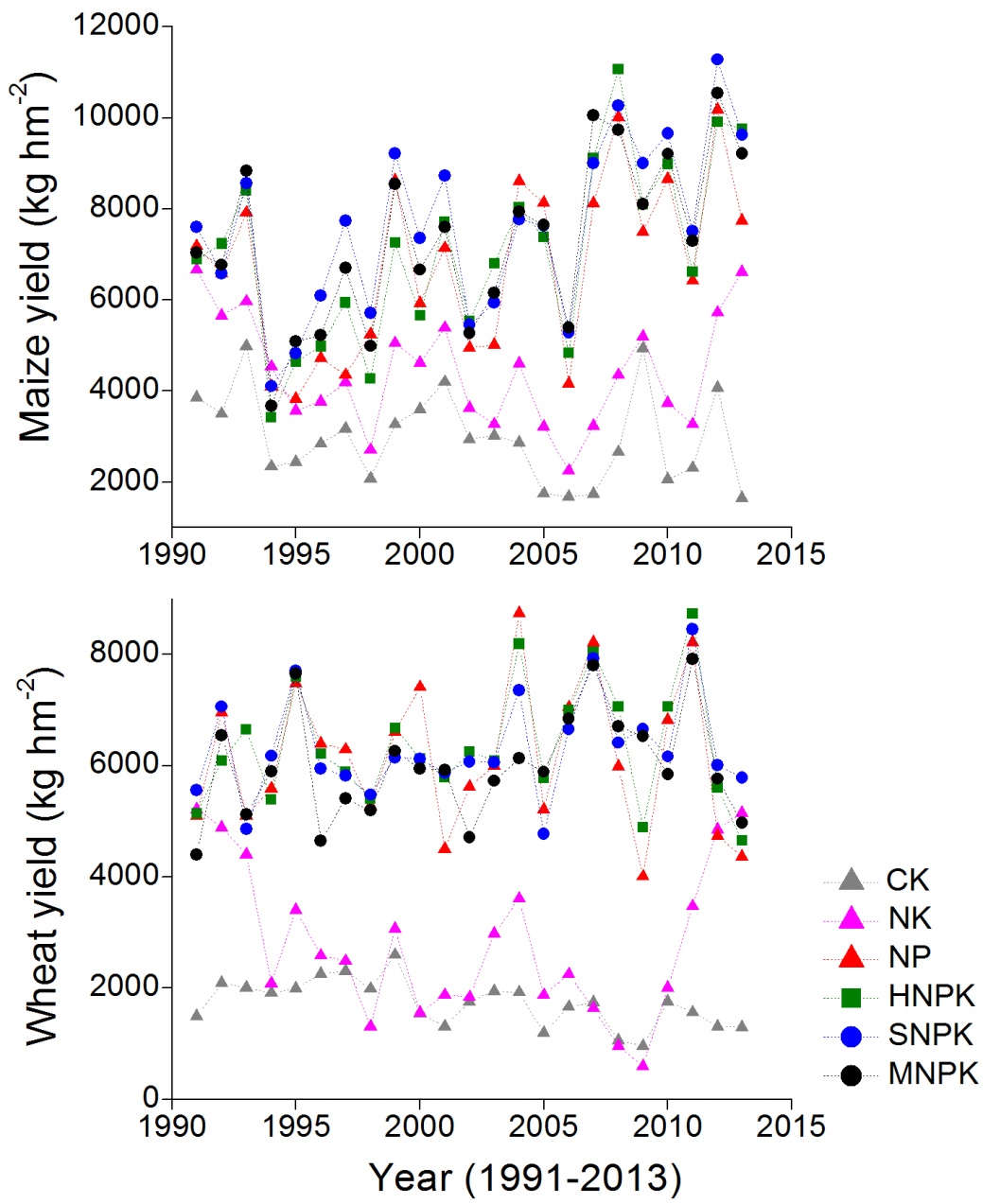


**Figure S1** Changes in crop yields for plots given different amendments and fertilizers during 1991‒2013. CK, unfertilized control; HNPK, high N and regular PK fertilizers; SNPK, maize straw plus NPK fertilizers; MNPK, organic manure plus NPK fertilizers.
